# Long-Term Health Associated with Small and Large for Gestational Age Births among Young Thai Adults

**DOI:** 10.3390/children9060779

**Published:** 2022-05-25

**Authors:** Alisha Suhag, Amaraporn Rerkasem, Kanokwan Kulprachakarn, Wason Parklak, Chaisiri Angkurawaranon, Kittipan Rerkasem, José G. B. Derraik

**Affiliations:** 1 The Healthy Lifespan Institute, University of Sheffield, Sheffield S10 2TN, UK; asuhag1@sheffield.ac.uk; 2 School of Health and Related Research, University of Sheffield, Sheffield S10 2TN, UK; 3 Research Institute for Health Sciences, Chiang Mai University, Chiang Mai 50200, Thailand; amaraporn.rer@gmail.com (A.R.); k_kulprachakarn@hotmail.com (K.K.); wason.p@cmu.ac.th (W.P.); 4 Department of Family Medicine, Faculty of Medicine, Chiang Mai University, Chiang Mai 50200, Thailand; chaisiri.a@cmu.ac.th; 5 Department of Surgery, Faculty of Medicine, Chiang Mai University, Chiang Mai 50200, Thailand; 6 Department of Paediatrics: Child and Youth Health, Faculty of Medical and Health Sciences, University of Auckland, Auckland 1142, New Zealand; 7 Department of Women’s and Children’s Health, Uppsala University, SE-751 85 Uppsala, Sweden; 8 Liggins Institute, University of Auckland, Auckland 1142, New Zealand

**Keywords:** AGA, appropriate for gestational age, birth weight, Chiang Mai, developmental origins of health and disease, DOHaD, LGA, obesity, overweight, SGA, small for gestational age

## Abstract

We examined the long-term health outcomes associated with being born small for gestational age (SGA) or large for gestational age (LGA). A total of 632 young adults aged ≈20.6 years were recruited from a longitudinal study (Chiang Mai, Thailand) in 2010: 473 born appropriate for gestational age (AGA), 142 SGA, and 17 LGA. The clinical assessments included anthropometry, blood pressure (BP), lipid profile, and an oral glucose tolerance test (OGTT). Young adults born SGA were 1.8 and 3.2 cm shorter than AGA (*p* = 0.0006) and LGA (*p* = 0.019) participants, respectively. The incidence of short stature was 8% among SGA compared with 3% in AGA and no cases among LGA participants, with the adjusted relative risk (aRR) of short stature among SGA 2.70 times higher than that of AGA counterparts (*p* = 0.013). SGA participants also had a 2 h glucose 7% higher than that of the AGA group (105 vs. 99 mg/dL; *p* = 0.006). Young adults born LGA had a BMI greater by 2.42 kg/m^2^ (*p* = 0.025) and 2.11 kg/m^2^ (*p* = 0.040) than those of SGA and AGA, respectively. Thus, the rate of overweight/obesity was 35% in the LGA group compared with 14.2% and 16.6% of SGA and AGA groups, respectively, with corresponding aRR of overweight/obesity of 2.95 (*p* = 0.011) and 2.50 (*p* = 0.017), respectively. LGA participants had markedly higher rates of BP abnormalities (prehypertension and/or hypertension) with an aRR of systolic BP abnormalities of 2.30 (*p* = 0.023) and 2.79 (*p* = 0.003) compared with SGA and AGA groups, respectively. Thai young adults born SGA had an increased risk of short stature and displayed some impairment in glucose metabolism. In contrast, those born LGA were at an increased risk of overweight/obesity and elevated blood pressure. The long-term follow-up of this cohort is important to ascertain whether these early abnormalities accentuate over time, leading to overt cardiometabolic conditions.

## 1. Introduction

Birth weight is an important indicator of prenatal health. Babies born at the extremes of the birth weight spectrum—small for gestational age (SGA) or large for gestational age (LGA) [1]—are at a greater risk of adverse health outcomes later in life (e.g., obesity, type-2 diabetes, and cardiovascular disease) [2,3]. Most of the evidence on the long-term outcomes associated with being born SGA or LGA comes from high-income countries (mainly from Caucasian populations in the USA and Europe [4,5]). However, the geographical, regional, and ethnic variations in maternal and perinatal characteristics may lead to SGA and LGA births [6]. For instance, maternal short stature in low- to middle-income countries appears to be a particularly important regional risk factor of SGA birth [7].

Given that Asia is inhabited by 60% of the world’s population, it is necessary to better understand the associations between being born SGA or LGA and the long-term health outcomes in this region. The countries with the highest prevalence of LGA (>10%) in Asia (i.e., Bangladesh, China, India, Japan, Thailand, and Vietnam) are also those traditionally characterised by low average birth weights and a high prevalence of SGA births [5]. This is because many Asian countries experience the additional burden of malnutrition [8]. However, there is a lack of studies examining long-term health outcomes among those born SGA or LGA in many of these countries. Previously, our research group briefly looked at the relationship between birth weight and the likelihood of metabolic syndrome in a subset of 393 young adults using body composition data from the longitudinal Chiang Mai Low Birth Study, finding no such association [9]. Here, we assessed the long-term health outcomes associated with SGA or LGA births across all participants with follow-up data from the same birth cohort, particularly the risk of short stature and obesity, since, to the best of our knowledge, these associations have not been previously examined in Thailand.

## 2. Materials and Methods

### 2.1. Ethics Approval

The Human Experimentation Committee at the Research Institute for Health Sciences (RIHES) at Chiang Mai University granted ethics approval for this study (#17/52). Written informed consent was obtained from all participants, and our study followed the relevant institutional and international principles as per the Declaration of Helsinki [10].

### 2.2. Study Design

Our study population included young adults followed up in 2010 at ≈20 years of age [11], who were the offspring born to mothers from the Chiang Mai Low Birth Weight Study in Northern Thailand in 1989/1990 [12]. The original study recruited 2184 women at ≤24 weeks of gestation from the Maternal-Child Health Care Center and Maharaj Nakorn Chiang Mai Hospital, the only public hospitals providing antenatal care in the province [12].

Demographic and clinical data were recorded in the original study, including birth parameters [13], maternal age, maternal alcohol intake and smoking during pregnancy, family income, and parents’ levels of education. Birth weights were transformed into z-scores according to the INTERGROWTH-21st standards [14], and participants then stratified into three groups: small for gestational age (SGA; <10th percentile); appropriate for gestational age (AGA; ≥10th percentile but <90th percentile); and large for gestational age (LGA; ≥90th percentile).

Following an overnight fast, clinical assessments were performed at the Research Institute for Health Science (RIHES) at Chiang Mai University. Anthropometric measurements were obtained with participants wearing light clothing and barefoot; weight was measured to the nearest 0.1 kg on calibrated electronic scales and height to the nearest 1 mm using a wall-mounted stadiometer. Short stature was defined as height <5th percentile for Thai adults as per Jordan et al. [15], equivalent to 149.3 cm for women and 159.9 cm for men. Body mass index (BMI) was calculated and the participant’s BMI status defined: underweight/normal weight <25 kg/m^2^; overweight ≥25 and <30 kg/m^2^; obesity ≥30 kg/m^2^. Venous blood samples (fasting) were drawn to measure lipids, glucose, and insulin, with insulin sensitivity estimated using the homeostatic model assessment of insulin resistance (HOMA-IR) [16]. Participants also underwent an oral glucose tolerance test (OGTT) after consuming a 250 mL solution containing 75 g of glucose, with a 2 h blood sample drawn to measure the glucose levels.

Blood pressure (BP) was measured twice on the left arm at rest, in a sitting position, using a digital sphygmomanometer (Terumo ES-P311; Terumo Corporation, Tokyo, Japan). Measurements were taken 5 min apart and their average recorded. Blood pressure abnormalities were categorised as: systolic prehypertension (SBP ≥130 and <140 mmHg), systolic hypertension (SBP ≥140 mmHg), diastolic prehypertension (DBP ≥85 and <90 mmHg), and diastolic hypertension (DBP ≥90 mmHg) [17]. In addition, abnormal SBP was classified as BP ≥130 mmHg and abnormal DBP as ≥85 mmHg. Maternal pregnancy-induced hypertension was defined as SBP ≥140 mmHg and/or DBP ≥90 mmHg, developed in a previously normotensive woman after 20 weeks of gestation and without proteinuria [18].

### 2.3. Statistical Analyses

Demographic characteristics in the three groups (SGA, AGA, and LGA) were compared with Fisher’s exact tests (categorical parameters) and one-way ANOVA or nonparametric Kruskal–Wallis tests (for continuous variables), as appropriate.

General linear regression models were used to examine the continuous outcomes, with unadjusted analyses initially performed. Analyses were subsequently run adjusting for important confounders: gestational age at birth and sex. Additional confounders were included according to the outcome of interest: for offspring height–mother’s height; for offspring weight and BMI–maternal BMI and smoking habit (never smoked, current smoker, and former smoker); and for offspring BP–mother’s pregnancy-induced hypertension (yes/no) [19].

Generalised linear models based on a Poisson distribution were used to estimate the unadjusted relative risks (RR) and adjusted relative risks (aRR) of short stature, overweight/obesity, abnormal SBP, and abnormal DBP. The models were similarly adjusted for appropriate confounders, as mentioned above.

Analyses were carried out using SAS v9.4 (SAS Institute, Cary, NC, USA). All statistical tests were two-tailed, with statistical significance maintained at the 5% level, with no adjustments for multiple comparisons, as per Rothman [20].

## 3. Results

### 3.1. Study Population

**Figure 1:**
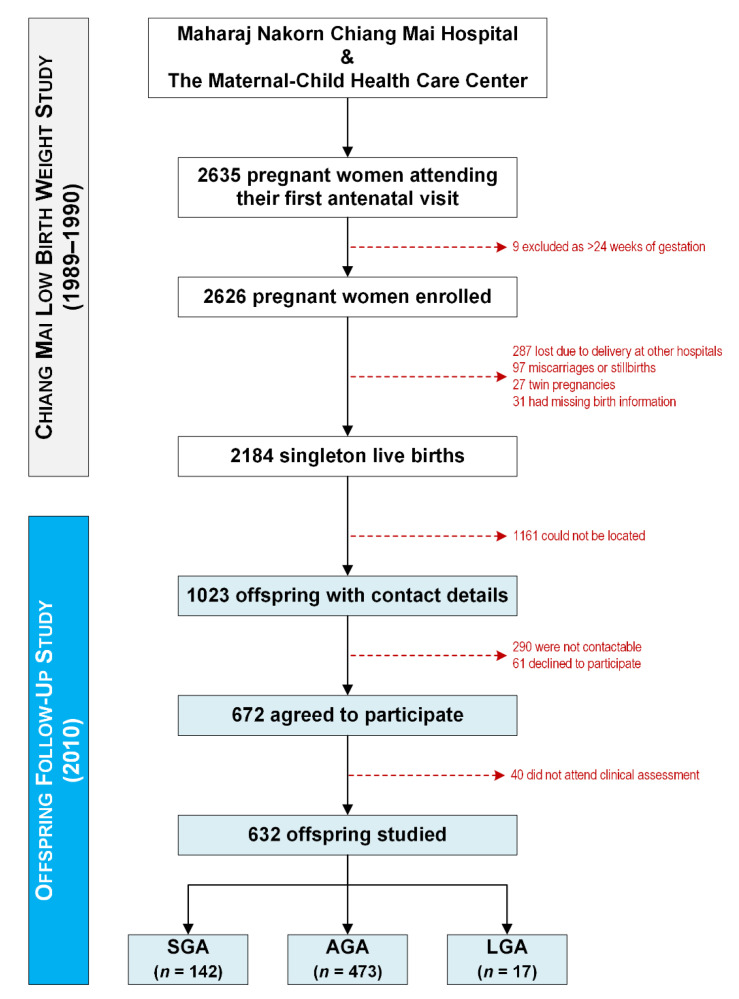
The recruitment of participants to the original Chiang Mai Low Birth Weight Study (1989–1990) and later on to the follow-up study on the offspring (2010).

There were 2184 children born in the original study, but 1552 were lost to follow-up; thus, our study included 632 young adults assessed at approximately 20.6 years of age (Figure 1). Our study population included 473 participants born AGA, 142 SGA, and 17 LGA (Table 1). Apart from the obvious differences in anthropometry at birth, LGA participants were born slightly earlier (*p* = 0.003) and to mothers with a higher average BMI (*p* = 0.0001), and in families with lower income (*p* = 0.027) compared with those born AGA (Table 1).

Our study participants were similar to the remainder of the original cohort [13], but the follow-up group had a greater proportion of women (54% vs. 44%; *p* < 0.001) and individuals born SGA (22.5% vs. 17.5%; *p* = 0.008), and a median family income 14% lower (*p* = 0.005) (Supplementary Table S1). Participants born SGA, LGA, and AGA who were captured in the follow-up study were also largely similar to those lost to follow-up (Supplementary Table S2); however, our current AGA population included proportionally more women (55.0% vs. 43.3%; *p* < 0.001) but fewer participants delivered by Caesarean section (7.0% vs. 11.1%; *p* = 0.015) (Supplementary Table S2). While the 17 LGA participants in the current study corresponded to approximately one-quarter (23%) of all individuals born LGA in the original study (*n* = 75), they were representative of those born LGA lost to follow-up (Supplementary Table S2).

### 3.2. Anthropometry

**Figure 2:**
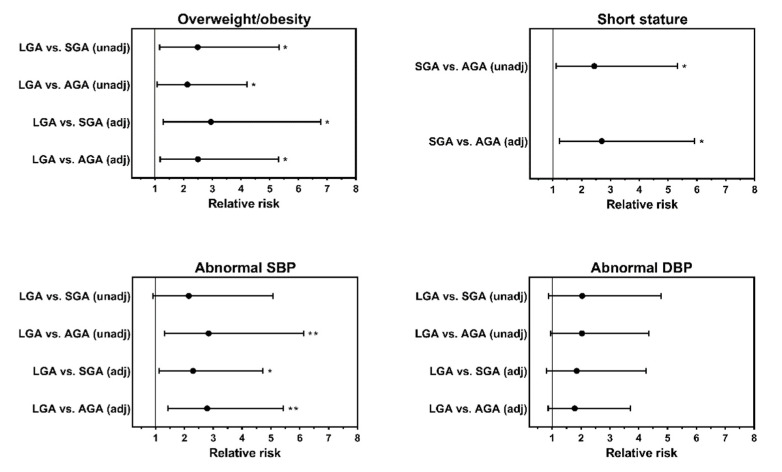
Adjusted relative risks of adverse anthropometric and blood pressure outcomes among young adults in Thailand according to their birth weight status. Data are relative risks or adjusted relative risks and respective 95% confidence intervals; * *p* < 0.05 and ** *p* < 0.01 for the difference between two given groups. AGA, appropriate-for-gestational-age; DBP, diastolic blood pressure; LGA, large for gestational age; SBP, systolic blood pressure; and SGA, small for gestational age. Overweight/obesity was defined as a body mass index ≥25 kg/m^2^; short stature was defined as height <5th percentile for Thai adults based on Jordan et al. [15], equivalent to 149.3 cm for women and 159.9 cm for men; abnormal SBP was defined as ≥130 mmHg and abnormal DBP as ≥85 mmHg.

Young Thai adults born SGA were 1.8 cm shorter than those born AGA (95% CI −2.8, −0.8 cm; *p* = 0.0006) and 3.2 cm than LGA (95% CI −5.9, −0.5 cm; *p* = 0.019) (Table 2). Furthermore, the incidence of short stature was 8% in the SGA group compared with 3% in the AGA group, with no participants recorded as such among those born LGA (Table 3). Therefore, the aRR of short stature in the SGA group was 2.70 (95% CI 1.23, 5.91; *p* = 0.013) times higher than that of AGA counterparts (Figure 2).

After adjustment for confounders (including maternal BMI), young adults born LGA had a BMI that was 2.42 kg/m^2^ (95% CI 0.30, 4.53 kg/m^2^; *p* = 0.025) and 2.11 kg/m^2^ (95% CI 0.10, 4.12 kg/m^2^; *p* = 0.040) higher than those of SGA and AGA, respectively (Table 2). Consequently, 35.3% of LGA participants had overweight/obesity compared with 14.2% and 16.6% of young adults born SGA and AGA, respectively (Table 3), with respective aRRs of 2.95 (95% CI 1.29, 6.78; *p* = 0.011) and 2.50 (95% CI 1.18, 5.31; *p* = 0.017) (Figure 2).

### 3.3. Cardiometabolic Outcomes

For the parameters associated with glucose metabolism, there were no differences in fasting glucose, fasting insulin, or HOMA-IR between groups (Table 2). However, the 2 h glucose during the OGTT was 7% higher in those born SGA than in the AGA group (*p* = 0.006; Table 2).

Adjusted analyses showed no differences in lipid profile or BP parameters between the three groups (Table 2). However, the rates of BP abnormalities (including prehypertension and/or hypertension) were markedly higher in the LGA group (Table 3); the aRR of SBP abnormalities among LGA was 2.30 (95% CI 1.12, 4.72; *p* = 0.023) and 2.79 (95% CI 1.43, 5.43; *p* = 0.003) in comparison with those born SGA and AGA, respectively (Figure 2).

## 4. Discussion

From our prospective cohort study, at approximately 20 years of age, Thai young adults born SGA had a higher risk of short stature, with some evidence suggestive of impairment in glucose metabolism compared with those born AGA. In contrast, those born LGA had higher BMI and were at increased risk of overweight/obesity and systolic BP abnormalities compared with the SGA and/or AGA groups.

While it seems that no studies have previously examined the long-term outcomes in association with SGA in Thailand, our findings are consistent with existing evidence showing persistent short stature as a common feature following SGA birth [21,22]. One study reported that the risk of short stature was five- to seven-fold higher in subjects born SGA [23]. Similarly, a systematic review found that the median prevalence of those born SGA who did not eventually reach normal adult height was 12.6% [24]. Our results are also corroborated by a regional cohort study in France reporting that 13.4% of 20–21-year-olds born SGA were short (i.e., >2 standard deviations below the mean height of controls) compared with only 2.6% of those born AGA [25]. Many children born SGA exhibit spontaneous catch-up growth, especially during the first years of life, enabling a progressive change in birth size and weight into the normal range [24,26]. While most children born SGA achieve an appropriate catch-up growth by two years of age, approximately 15% do not, often experiencing poor growth [22]. Parental height is a strong genetic predictor of adult short stature in those born SGA [26]. Still, the mechanisms underlying the absence or presence of catch-up growth (and therefore persistent short stature) are poorly understood [27].

We also observed increased 2 h glucose concentrations (during the OGTT) among those born SGA compared with the AGA group, suggestive of some impairment in glucose metabolism. Our findings corroborate previous observations of higher serum glucose concentrations during an OGTT in another group of 20-year-olds born SGA compared with age-matched subjects born AGA [28], with other studies reporting similar findings starting from childhood [29,30]. Furthermore, a systematic review showed that low birth weight was associated with adverse glucose metabolism in both children and adults [31].

Despite the relatively small number of participants born LGA in our cohort, we observed increased risks of overweight/obesity compared with those born SGA or AGA, corroborating evidence on Caucasians [32,33,34,35]. For example, a large study on Swedish women found that those born LGA were 50% more likely to have obesity in adulthood than those born AGA [32]. Our findings also agree with systematic reviews and meta-analyses showing an association between high birth weight and increased risk of obesity from childhood to early adulthood [35,36,37]. Notably, our findings of increased BMI and overweight/obesity risk persisted even after adjustment for maternal BMI (which is an important determinant of high birth weight and later obesity risk [38]), indicating that factors other than genetics may be at play, including epigenetic changes in utero. Similar factors likely explain our observations of increased blood pressure abnormalities in individuals born LGA. However, unlike the data on obesity risk, the evidence on associations between LGA (or high birth weight) and blood pressure later in life is conflicting [39,40,41].

A limitation of our study was the lack of data on dietary intake and physical activity, which are relevant factors for key health outcomes such as the risk of obesity and elevated blood pressure. In addition, a relatively small proportion of our study population was born LGA (*n* = 17; ≈3%), which made up 23% of all those born LGA in the original study. Although individuals born LGA who were lost to follow-up had similar demographic and birth characteristics to those captured in the present study, this small group limits our ability to extrapolate our findings on LGA to the general Thai population. Lastly, although our assessments of height outcomes accounted for maternal stature, paternal height was not recorded. Thus, the participants’ genetic height potential (target height) could not be estimated [42,43]; accounting for paternal stature could have affected our findings on short stature, particularly among men. However, it is likely that our ethnically homogeneous cohort would have mitigated this issue to a large extent. Nonetheless, a key strength of our study is that, to the best of our knowledge, this is the first long-term follow-up of a prospective cohort examining outcomes specifically among those born SGA and LGA in Thailand. Further, we also examined a comprehensive range of outcomes, including anthropometric and cardiometabolic parameters.

## 5. Conclusions

Our study showed that Thai young adults born SGA had an increased risk of short stature in later life, also displaying some impairment in glucose metabolism. In contrast, at the other end of the birth weight spectrum, young adults born LGA were at an increased risk of overweight/obesity and elevated blood pressure. Thus, long-term follow-up of this cohort will be important to corroborate these findings, ideally including the recapture of more participants born LGA who were absent from our 20-year follow-up study. This would help ascertain whether these observed early abnormalities accentuate over time, leading to overt cardiometabolic conditions.

## Figures and Tables

**Table 1:** Demographic and birth characteristics of our study population stratified by birth weight status.

Characteristic		SGA	AGA	LGA
*n*		142 (22.5%)	473 (74.8%)	17 (2.7%)
Sex	Male	71 (50%)	212 (45%)	8 (47%)
	Female	71 (50%)	261 (55%)	9 (53%)
Delivery	Vaginal delivery	122 (86%)	439 (93%)	14 (82%)
	Caesarean section	20 (14%)	34 (7%)	3 (18%)
Age (years)		20.5 ± 0.5	20.6 ± 0.5	20.6 ± 0.5
Birth weight (kg)		2.55 ± 0.27	3.08 ± 0.36	3.75 ± 0.66
Birth weight Z-score		−1.78 ± 0.40	−0.24 ± 0.61	1.69 ± 0.33
Birth length (cm)		47.3 ± 2.1	49.2 ± 4.3	51.6 ± 4.2
Birth length Z-score		−1.18 ± 0.98	0.21 ± 1.15	1.98 ± 1.22
Gestational age (weeks)		39.6 ± 1.4	39.1 ± 1.7	38.4 ± 2.8
Maternal age at childbirth (years)		26.5 ± 4.5	26.2 ± 4.7	28.5 ± 4.2
Maternal BMI (kg/m^2^)		20.62 ± 2.33	21.51 ± 2.51	22.56 ± 2.95
Maternal BMI status	Underweight/normal weight	135 (95%)	432 (91%)	12 (71%)
	Overweight/obesity	7 (5%)	41 (9%)	5 (29%)
Maternal education	Less than high school	109 (89%)	366 (91%)	12 (92%)
	High school or greater	14 (11%)	37 (9%)	1 (8%)
Paternal education	Less than high school	97 (80%)	329 (81%)	10 (77%)
	High school or greater	25 (20%)	76 (19%)	3 (23%)
Family income (baht per month) ^1^		2300 [1500, 3500]	2700 [1675, 4400]	1700 [1000, 2320]

Data are the means ± SD, *n* (%), or medians [quartile 1, quartile 3], as appropriate. AGA, appropriate for gestational age; BMI, body mass index; LGA, large for gestational age; and SGA, small for gestational age. ^1^ Income recorded at maternal recruitment to the original study in 1989–1990.

**Table 2:** Anthropometric and cardiometabolic outcomes in our cohort of young adults in Thailand according to birth weight status.

		Unadjusted			Adjusted		
		SGA	AGA	LGA	SGA	AGA	LGA
Anthropometry	Height (cm)	162.8 (161.4, 164.1)	164.0 (163.3, 164.8)	165.6 (161.6, 169.5)	162.8 (161.9, 163.7) ***†	164.6 (164.1, 165.1)	166.1 (163.5, 168.6)
	Weight (kg)	55.6 (53.3, 57.9) ††	57.8 (56.5, 59.1) †	65.0 (58.3, 71.6)	56.9 (54.4, 59.4) ††	59.0 (57.2, 60.9) †	65.47 (59.3, 71.5)
	BMI (kg/m^2^)	20.86 (20.15, 21.57) ††	21.36 (20.97, 21.75) †	23.72 (21.68, 25.77)	21.06 (20.37, 21.75) †	21.36 (20.98, 21.73) †	23.48 (21.50, 25.46)
Glucose homeostasis	Fasting glucose (mg/dL)	83 (82, 85)	83 (82, 83)	84 (80, 88)	83 (82, 85)	83 (82, 83)	84 (80, 89)
	Fasting insulin (mIU/L)	7.8 (6.9, 8.7)	7.2 (6.7, 7.6)	9.6 (6.9, 13.1)	7.8 (7.0, 8.7)	7.1 (6.7, 7.5)	9.5 (6.9, 13)
	120-min glucose (mg/dL)	105 (101, 110) **	99 (97, 101)	96 (86, 107)	105 (101, 109) **	99 (97, 101)	96 (87, 107)
	HOMA-IR	1.60 (1.42, 1.79)	1.47 (1.37, 1.56)	1.99 (1.43, 2.76)	1.60 (1.42, 1.79)	1.45 (1.36, 1.55)	1.97 (1.42, 2.74)
Blood pressure	Systolic (mmHg)	115 (113, 117)	114 (113, 116)	117 (111, 123)	116 (114, 119)	116 (113, 118)	117 (112, 123)
	Diastolic (mmHg)	75 (73, 76)	73 (73, 74) †	79 (74, 84)	76 (74, 78)	75 (73, 77)	80 (75, 85)
Lipid profile	Total cholesterol (mg/dL)	169 (163, 174)	168 (165, 172)	175 (158, 191)	168 (162, 174)	168 (165, 172)	175 (159, 192)
	HDL (mg/dL)	56 (53, 58)	57 (55, 58)	57 (50, 64)	56 (53, 58)	56 (55, 58)	57 (50, 64)
	LDL (mg/dL)	96 (91, 101)	95 (93, 98)	99 (85, 113)	96 (91, 101)	96 (93, 98)	100 (86, 114)
	Triglycerides (mg/dL)	76 (70, 83)	76 (72, 79)	82 (65, 104)	76 (70, 83)	76 (73, 80)	83 (66, 105)

AGA, appropriate for gestational age; BMI, body mass index; HDL, high-density lipoprotein cholesterol; HOMA-IR, homeostatic model assessment of insulin resistance; LDL, low-density lipoprotein cholesterol; LGA, large for gestational age; SGA, small for gestational age. Data are means and respective 95% confidence intervals. ** *p* < 0.01 and *** *p* < 0.001 in comparison with AGA; † *p* < 0.05 and †† *p* < 0.01 in comparison with the LGA group.

**Table 3:** Adverse anthropometric and blood pressure outcomes among young adults in Thailand according to their birth weight status.

			SGA	AGA	LGA
Anthropometry	*n*		141	470	17
	BMI status	Underweight/normal weight	121 (85.8%)	392 (83.4%)	11 (64.7%)
		Overweight	12 (8.5%)	54 (11.5%)	4 (23.5%)
		Obesity	8 (5.7%)	24 (5.1%)	2 (11.8%)
		Overweight/obesity	20 (14.2%)	78 (16.6%)	6 (35.3%)
	Height	Normal stature	130 (92.2%)	455 (96.8%)	17 (100%)
		Short stature	11 (7.8%)	15 (3.2%)	nil
Blood pressure	*n*		139	463	17
	Systolic	Normotension	120 (86.3%)	415 (89.6%)	12 (70.6%)
		Prehypertension	15 (10.8%)	30 (6.5%)	4 (23.5%)
		Hypertension	4 (2.9%)	18 (3.9%)	1 (5.9%)
		Abnormal	19 (13.7%)	48 (10.4%)	5 (29.4%)
	Diastolic	Normotension	119 (85.6%)	396 (85.5%)	12 (70.6%)
		Prehypertension	11 (7.9%)	43 (9.3%)	4 (23.5%)
		Hypertension	9 (6.5%)	24 (5.2%)	4 (5.9%)
		Abnormal	20 (14.4%)	67 (14.5%)	5 (29.4%)

Data are *n* (%). AGA, appropriate for gestational age; BMI, body mass index; LGA, large for gestational age; SGA, small for gestational age. BMI status was defined as: underweight/normal weight <25 kg/m^2^; overweight ≥25 but <30 kg/m^2^; obesity ≥30 kg/m^2^; and overweight/obesity ≥25 kg/m^2^. Short stature was defined as height <5th percentile for Thai adults based on Jordan et al. [15], equivalent to 149.3 cm for women and 159.9 cm for men. Blood pressure (BP) abnormalities were defined as follows: systolic prehypertension, SBP ≥130 but <140 mmHg; systolic hypertension, SBP ≥140 mmHg; abnormal systolic BP, SBP ≥130 mmHg; diastolic prehypertension, DBP ≥85 but <90 mmHg; diastolic hypertension, DBP ≥90 mmHg; abnormal diastolic BP, SBP ≥85 mmHg.

## Data Availability

The anonymised data on which this manuscript is based can be made available to other investigators upon *bona fide* request and following all the necessary approvals (including ethics approval) of the detailed study proposal and statistical analyses plan. Any queries should be directed to Professor Kittipan Rerkasem (kittipan@rihes.org).

## Supplementary Materials

The following supporting information can be downloaded at: https://www.mdpi.com/article/10.3390/children9060779/s1, Table S1: Demographic and birth characteristics among participants from the Chiang Mai Low Birth Weight Study who were Lost to follow-up or captured in the Follow-up Study at ≈20 years of age; Table S2: Demographic and birth characteristics among participants from the Chiang Mai Low Birth Weight Study who were Lost to follow-up or captured in the Follow-up Study at ≈20 years of age according to their birth weight status.
