# Supplementary material for: Long-Term Health Associated with Small and Large for Gestational Age Births among Young Thai Adults
Source: Children (Basel). 2022 May 25;9(6):779. doi: 10.3390/children9060779 (PMC9221860; doi:10.3390/children9060779)
Supplement: Supplementary file 1 [file children-09-00779-s001.zip › children-1603946-supplementary.pdf]

Supplementary Table S1

Demographic and birth characteristics among participants from the Chiang Mai Low Birth Weight Study who were Lost to follow-up or captured in the Follow-up Study at ≈20 years of age.

Characteristic	Levels	Lost to follow-up	Follow-up study
n		1552	632
**Sex**	**Males**	876 (56.4%)	292 (46.2%) ***
	**Females**	676 (43.6%)	340 (53.8%)
**Birth weight *z*-score** [a]		-0.32 ± 0.92	-0.46 ± 0.92 **
**Birth weight status** [a]	**SGA**	271 (17.5%)	142 (22.5%) **
	**AGA**	1223 (78.8%)	473 (74.8%) *
	**LGA**	58 (3.7%)	17 (2.7%)
**Gestational age (weeks)**		38.8 ± 2.0	39.0 ± 1.7 *
**Delivery by caesarean section**		57 (9.0%)	176 (11.3%)
**Maternal age at childbirth (years)**		25.3 ± 4.6	26.3 ± 4.6 ***
**Maternal BMI (kg/m^2)** [b]		21.51 ± 2.86	21.34 ± 2.51
**Maternal PIH** [c]		40 (2.6%)	25 (4.0%)
**Maternal smoking at pregnancy**		14 (0.9%)	5 (0.8%)
**Maternal alcohol consumption during pregnancy**		11 (0.7%)	7 (1.1%)
**Maternal education** [d]	**Less than high school**	1127 (89.2%)	491 (90.3%)
	**High school or greater**	136 (10.8%)	53 (9.7%)
**Paternal education**	**Less than high school**	1036 (82.1%)	439 (80.7%)
	**High school or greater**	226 (17.9%)	105 (19.3%)
**Family income (baht per month)** [e]		2918 [1800, 4500]	2500 [1563, 4200] **

Table adapted from Rerkasem et al. Caesarean delivery is associated with increased blood pressure in young adult offspring. Sci Rep 2021;11(1):10201.

Continuous data are mean ± standard deviation or median [quartile 1, quartile 3], as appropriate; categorical data are *n* (%).

AGA, appropriate-for-gestational-age (birth weight ≥10th but <90th percentile); BMI, body mass index; LGA, large-for-gestational-age (birth weight ≥90th percentile); PIH, pregnancy-induced hypertension; SGA, small-for-gestational-age (birth weight <10th percentile).

[a] Data on gestational age at birth were missing for 4 participants, so their birth weight *z*-scores could not be calculated.

[b] BMI recorded at the first antenatal visit in the original study in 1989–1990.

[c] PIH was defined as a systolic blood pressure ≥140 mmHg and/or diastolic blood pressure ≥90 mmHg developed after 20 weeks of gestation without proteinuria, in a previously normotensive woman.

[d] There were missing data on the highest levels of education, so that the available sample sizes for the Follow-up and Lost to follow-up groups were 544 (86.1%) and 1263 (81.4%) for maternal education, respectively, and 544 (86.1%) and 1262 (81.3%) for paternal education.

[e] Income recorded at the time of maternal recruitment to the original study in 1989–1990 (i.e., not adjusted for inflation); the available sample sizes were 542 (85.8%) for the Follow-up group and 1230 (79.3%) for those Lost to follow-up.

*p<0.05, **p<0.01, and ***p<0.001 for comparisons between the Lost to follow-up and Follow-up study participants; p-values were derived from Chi-squared or Fisher's exact tests for categorical parameters, non-parametric Kruskal-Wallis tests for family income, and two-sample t-tests for all other continuous variables.

Supplementary Table S2

Demographic and birth characteristics among participants from the Chiang Mai Low Birth Weight Study who were Lost to follow-up or captured in the Follow-up Study at ≈20 years of age according to their birth weight status.

		SGA	SGA	AGA	AGA	LGA	LGA
**Characteristic**	**Levels**	**Lost to follow-up**	**Follow-up study**	**Lost to follow-up**	**Follow-up study**	**Lost to follow-up**	**Follow-up study**
n		271	142	1223	473	58	17
**Sex (females)**		119 (43.9%)	71 (50.0%)	530 (43.3%)	261 (55.0%) ***	27 (46.6%)	9 (53.0%)
**Birth weight *z*-score**		-1.74 ± 0.39	-1.78 ± 0.40	-0.22 ± 0.63	-0.24 ± 0.61	1.76 ± 0.44	1.69 ± 0.33
**Gestational age (weeks)**		39.6 ± 1.6	39.6 ± 1.4	39.0 ± 1.8	39.1 ± 1.7	38.2 ± 2.6	38.4 ± 2.8
**Preterm birth**		15 (5.5%)	4 (2.8%)	72 (5.9%)	28 (5.9)	8 (13.8%)	2 (11.8%)
**Delivery by caesarean section**		26 (9.6%)	20 (14.0%)	136 (11.1%)	34 (7.0%) *	14 (24.1%)	3 (18.0%)
**Maternal age at childbirth (years)**		25.4 ± 4.7	26.5 ± 4.5 *	25.2 ± 4.6	26.2 ± 4.7 ***	26.5 ± 4.7	28.5 ± 4.2
**Maternal BMI (kg/m^2) [a]**		21.0 ± 2.7	20.6 ± 2.3	21.6 ± 2.8	21.5 ± 2.5	23.3 ± 3.4	22.5 ± 2.9
**Maternal PIH [b]**		9 (3.3%)	11 (7.7%)	29 (2.4%)	11 (2.3%)	2 (3.4%)	3 (17.6%)
**Maternal education**	**Less than high school**	212 (93.8%)	109 (88.6%)	878 (88.2%)	370 (90.7%)	37 (88.1%)	12 (92.3%)
	**High school or greater**	14 (6.2%)	14 (11.4%)	117 (11.8%)	38 (9.3%)	5 (11.9%)	1 (7.7%)
**Paternal education**	**Less than high school**	186 (82.7%)	97 (79.7%)	817 (82.1%)	329 (81.1%)	33 (78.6%)	10 (77.0%)
	**High school or greater**	39 (17.3%)	25 (20.3%)	178 (17.9%)	76 (18.9%)	9 (21.4%)	3 (23.0%)
**Family income (baht per month) [c]**		2500 [1500, 4000]	2300 [1500, 3500]	3000 [1872, 4530]	2700 [1675, 4400] *	2400 [1950, 4600]	1700 [1000, 2320]

Continuous data are means ± SD or median [quartile 1, quartile 3], as appropriate; categorical data are *n* (%).
AGA, appropriate-for-gestational-age (birth weight ≥10th but <90th percentile); BMI, body mass index; LGA, large-for-gestational-age (birth weight ≥90th percentile); PIH, pregnancy-induced hypertension; SGA, small-for-gestational-age (birth weight <10th percentile).
[a] Defined as a live birth <37 weeks of gestation.
[b] BMI recorded at the first antenatal visit in the original study in 1989–1990.
[c] PIH was defined as a systolic blood pressure ≥140 mmHg and/or diastolic blood pressure ≥90 mmHg developed after 20 weeks of gestation without proteinuria, in a previously normotensive woman.
[d] Income recorded at the time of maternal recruitment to the original study in 1989–1990 (i.e., not adjusted for inflation).
*p<0.05 and ***p<0.001 for comparisons between the Lost to follow-up and Follow-up study participants within a given birth weight status; p-values were derived from Chi-squared or Fisher's exact tests for categorical parameters, non-parametric Kruskal-Wallis tests for family income, and two-sample t-tests for all other continuous variables.

Suhag A, Rerkasem A, Kulprachakarn K, Parklak W, Angkurawaranon C, Rerkasem K, Derraik JGB. Long-term health associated with small and large for gestational age births among young Thai adults. *Children* 2022.
